# Exogenously Supplemented Proline and Phenylalanine Improve Growth, Productivity, and Oil Composition of Salted Moringa by Up-Regulating Osmoprotectants and Stimulating Antioxidant Machinery

**DOI:** 10.3390/plants11121553

**Published:** 2022-06-11

**Authors:** Amira K. G. Atteya, Rasha S. El-Serafy, Khaled M. El-Zabalawy, Abeer Elhakem, Esmail A. E. Genaidy

**Affiliations:** 1 Horticulture Department, Faculty of Agriculture, Damanhour University, Damanhour 22516, Egypt; 2 Horticulture Department, Faculty of Agriculture, Tanta University, Tanta 31527, Egypt; 3 Environment and Bio-Agriculture Department, Faculty of Agriculture, Al-Azhar University, Cairo 11651, Egypt; khaled_mohamed@azhar.edu.eg; 4 Department of Biology, College of Sciences and Humanities, Prince Sattam Bin Abdulaziz University, Al-Kharj 11942, Saudi Arabia; a.elhakem@psau.edu.sa; 5 Pomology Department, National Research Centre, Giza 12622, Egypt; esmail_nrc@yahoo.com

**Keywords:** salinity, moringa, proline, abiotic stress, fatty acids, oleic acid, linoleic acids

## Abstract

Salinity is linked to poor plant growth and a reduction in global food output. Therefore, there is an essential need for plant adaptation and mitigation of salinity stress conditions. Plants combat salinity stress influences by promoting a set of physiological, biochemical, and molecular actions. Tremendous mechanisms are being applied to induce plant stress tolerance, involving amino acid application. For evaluating the growth and productivity of *Moringa oleifera* trees grown under salt stress conditions, moringa has been cultivated under different levels of salinity and subjected to a foliar spray of proline (Pro) and phenylalanine (Phe) amino acids. Moringa plants positively responded to the lowest level of salinity as the leaves, inflorescences, seeds, and oil yields have been increased, but the growth and productivity slightly declined with increasing salinity levels after that. However, Pro and Phe applications significantly ameliorate these effects, particularly, Pro-treatments which decelerated chlorophyll and protein degradation and enhanced vitamin C, polyphenols, and antioxidant activity. A slight reduction in mineral content was observed under the high levels of salinity. Higher osmoprotectants (proline, protein, and total soluble sugars) content was given following Pro treatment in salted and unsalted plants. A significant reduction in oil yield was obtained as affected by salinity stress. Additionally, salinity exhibited a reduction in oleic acid (C18:1), linoleic (C18:2), and linolenic (C18:3) acids, and an increase in stearic (C18:0), palmitic (C16:0), eicosenoic (C20:2), and behenic (C22:0) acids. Generally, Pro and Phe treatments overcome the harmful effects of salinity in moringa trees by stimulating the osmoprotectants, polyphenols, and antioxidant activity, causing higher dry matter accumulation and better defense against salinity stress.

## 1. Introduction

*Moringa oleifera* belongs to the Moringaceae family, native to northwest India and widely cultivated in Central Africa, Asia, and America [1,2]. It is called a horseradish tree or a drumstick tree. Since the nutritional value, carbohydrates, essential amino acids, protein, and vitamins, as well as the mineral content in all components of the moringa tree, are edible, they are utilized in a variety of uses, including livestock feed, green manure, cleaning materials, biogas, and more [3,4,5]. The moringa tree components of leaves, inflorescences, seeds, oils, and roots can be used to cure a range of diseases, such as lung allergies, skin infections, and high blood pressure [6]. Many studies were performed on moringa tree leaf production under normal conditions. However, few studies have been conducted about Moringa trees’ leaf and oil productivity under stress conditions, including salinity.

Despite the vital role of water in all metabolic activities occurring in the plant cell, water shortages are becoming a global issue with escalating severity [7]. Therefore, using lower-quality water, such as saline water, is essential to compensate for water shortages. Salinity is one of the most important abiotic stressors that plants face, decreasing crop productivity and altering their physiological and morphological characteristics [8]. Salts impact plant growth through the changes in the imbalance of hormones, ionic toxicity, osmotic exertion, nutrient motivation reduction, and ROS production [9]. Therefore, salinity stress causes plants to wilt, dry, and die. Under salinity stress, plants acquire osmotically active synthesis named osmoprotectants in response to osmotic stress to reduce the osmotic potential. The osmoprotectants’ principal role is to maintain cell turgor, which serves as a driving force for the absorption of water and can also serve as free-radical scavengers [10,11]. Proline, protein, total soluble sugars, and amino acids are osmoprotectants found in plants as affected by salt stress [12,13]. Nowadays, a variety of organic molecules are used to ameliorate the harmful effects of salinity stress on plants, including amino acids [14].

Amino acids are essential for the biosynthesis of proteins, enzymes, and nitrogen-containing molecules that are important for plant development and defense. Proline (Pro) is an amino acid that has a vital role in plant tolerance under environmental stresses. It prevents protein oxidation, reduces lipid peroxidation, and preserves cell membranes and structures. Moreover, Pro is a source of nitrogen and energy [15]. The endogenous levels of Pro have been elevated under oxidative stress as a reaction to ROS generation, subsequently keeping enzymes from oxidation [16]. Under saline conditions, Vinocur and Altman [17] noticed that proline found in the cytosol contributed to osmotic stress, improving plant tolerance. Proline applications enhanced plants’ photosynthetic activity under saline conditions [18].

Phenylalanine (Phe) is an amino acid essential for plant growth and development and acts as a precursor of many molecules important in plant development, reproduction, and defense against abiotic and biotic stressors, including phenylpropanoids, flavonoids, anthocyanins, lignin, tannins, and salicylate [19]. In woody plants, Phe has a vital role in lignin synthesis during wood development, as phenylalanine ammonia-lyase (PAL) stimulates the transmutation of Phe to ammonium and trans-cinnamic acid in the vascular cells [19], which is then re-used for amino acid biosynthesis [20]. Phenylalanine alleviates the harmful impacts of salinity, and increased proteins, saccharides, and proline concentrations [21,22].

Hence, the current investigation was executed to shed more light on the ameliorative role of proline and phenylalanine on the productivity of leaves, seeds, and oil, as well as the oil composition, physiochemical, and antioxidant activities of Moringa olivera L. grown under saline conditions.

## 2. Materials and Methods

### 2.1. Location and Plant Materials

This pot experiment was undertaken at the Faculty of Agriculture, Damanhour University, Damanhour, Egypt during the two successive seasons of 2018/2019 and 2019/2020 under field conditions. *Moringa oleifera* seeds were obtained from the National Research Centre, Egypt, and sown in a 20 cm plastic pot (three seeds per pot) filled with clay loam soil on 1 February 2018 and 2019. The uniform seedlings (20-day old) were selected to be one seedling per pot. The physical and chemical properties of the water irrigation and soil samples were determined according to Jackson [23] and Cottenie [24] and are shown in Table 1 and Table 2.

### 2.2. Salinity Treatments

After seed sowing, the seeds were irrigated with tap water for three day intervals, and then the irrigation was continued once or twice a week until 50 days from sowing. Saline water treatments were started 50 days after sowing with concentrations of 0 (unsalted), 1000 (15.6 mM), 2000 (31.2 mM), 3000 (46.9 mM), and 4000 ppm (62.5 mM) of NaCl, twice a week. The unsalted plants were irrigated with tap water. After every four irrigation times with saline water, one time of tap water was used to avoid the accumulation of salt in the soil of pots.

### 2.3. Amino Acid Applications

Moringa plants were foliar sprayed with Pro and Phe amino acids (Sigma Aldrich, Burlington, MA, USA). The plants were divided into three groups: the first group received distilled water foliar application (without AA), the second group received 50 ppm of Pro solution, while the third group received 50 ppm of Phe solution. Amino acid preparation was done as follows: 50 mg of both Pro and Phe were dissolved separately in a liter of distilled water. The first foliar application was done five days before saline water treatments were applied and repeated every 15 days until pods ripped using a manual sprayer. Without AA plants were sprayed with distilled water. The pot’s soil was covered with a plastic sheet during spraying.

### 2.4. Experimental Layout

This pot experiment was designed in a factorial design with two factors in a randomized complete block design arrangement; salinity levels (5 levels) are the first factor, while amino acids (3 treatments) are the second factor. The experiment consists of 15 treatments; each treatment contains 3 replicates (10 pots/ replicate).

### 2.5. Growth and Yield Component Traits

For growth and yield traits determination, 14 months after sowing, five pots were randomly collected from each replicate to estimate the following growth parameters: plant height (cm), stem diameter (mm), leaves number tree^−^^1^, leaves fresh weight tree^−^^1^ (g), leaves dry weight tree^−^^1^ (g), inflorescences number tree^−^^1^, pods number inflorescence^−^^1^, pods number tree^−^^1^, pod weight (g), the yield of mature pod tree^−^^1^ (g), number of seeds per pod, seed weight (g), and seeds yield tree^−^^1^ (g).

### 2.6. Biochemical Analysis

#### 2.6.1. Total Chlorophyll

A SPAD-502 Chlorophyll Meter (Minolta Camera Co., Ramsey, NJ, USA) was used for total chlorophyll content evaluation (SPAD unit).

#### 2.6.2. Polyphenols Determination

Total phenols (mg Gallic g^−^^1^ DW) in moringa leaves were estimated as previously described by El-Serafy [25] and mentioned by Singleton and Rossi [26]. Flavonoids level in the leaves was determined colorimetrically using Kim [27] method (mg RUT g^−^^1^ DW).

#### 2.6.3. Osmoprotectants Estimation

Free proline content in moringa leaves was estimated using the Bates [28] procedure. Soluble protein contents (mg g^−^^1^) of the leaves were assessed using the Folin Ciocalteu reagent, as described by Lowry [29]. Total soluble sugars (TSS) in moringa leaves were determined according to the Herbert [30] procedure.

#### 2.6.4. Antioxidants and Vitamin C Measurements

The antiradical activity of leaves was assessed using the Stable 2,2-diphenyl-1-picrylhydrazyl radical (DPPH) according to the method of Brand-Williams [31]. The extract concentration (µg ml^−^^1^) providing 50% of antioxidant activities (IC50) was calculated by plotting in a graph inhibition percentage against extract concentration. Vitamin C in moringa leaves (mg g^−^^1^ DW) was determined according to A.O.A.C. [32] method.

#### 2.6.5. Mineral Determination

Leaves P content was determined colorimetrically as reported by Jackson [23], while K content was measured using atomic absorption spectrophotometry (Bk-AA4530f, China) according to the Ghosh and Paul’s [33] method.

### 2.7. Oil Estimation

The oil extracted from moringa seeds was estimated as the method of A.O.A.C. [32] and stored at 4 °C for GC-Mass analysis, and oil percent and oil yield tree^−^^1^ (mL) were calculated.

### 2.8. GC-MS Analysis

Fatty acid methyl esters were prepared following methanolic sulfuric acid and characterized by gas chromatography-mass spectrometry. The GC-MS analysis was performed at the Department of Medicinal and Aromatic Plants Research, National Research Center. Oil samples were analyzed using GC Ultra Gas Chromatographs (Thermo Scientific Corp., Waltham, MA, USA), coupled with a Thermo mass spectrometer detector (ISQ Single Quadrupole Mass Spectrometer). The GC-MS system was equipped with a TG-WAX MS column (30 m × 0.25 mm i.d., 0.25 μm film thickness). Analyses were carried out using helium as carrier gas at a flow rate of 1.0 mL min^−1^ and a split ratio of 1:10 using the following temperature program: 40 °C for 1 min; rising at 4.0 °C min^−1^ to 160 °C and held for 6 min; rising at 6 °C min^−1^ to 210 °C and held for 1 min. The injector and detector were held at 210 °C. Diluted samples (1:10 hexane, *v*/*v*) of 1 μL of the mixtures were always injected. Mass spectra were obtained by electron ionization at 70 eV, using a spectral range of *m*/*z* 40–450.

### 2.9. Statistical Analysis

The data collected were statistically analyzed by the MSTAT program, Bartlett’s test was used for assessing homogeneity variances, and a combined analysis was done. A Duncan multiple test (at 5% probability) was used for estimating the significant differences among mean values [34]. The results are presented as the average means of the two seasons ± standard error (SE).

## 3. Results

### 3.1. Growth and Yield Traits

**Figure 1:**
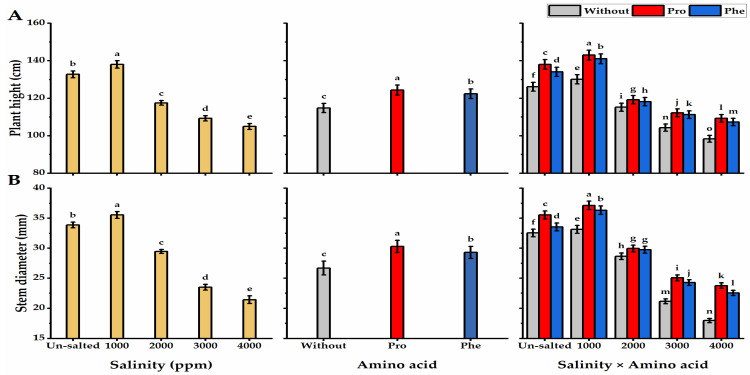
Influence of exogenous spray treatments of proline (Pro) or phenylalanine (Phe) on (**A**) plant height and (**B**) stem diameter of moringa trees cultivated under different levels of salinity. Data are mean value ± SE. Bars with different letters are significantly different at *p* ≤ 0.05 level.

**Figure 2:**
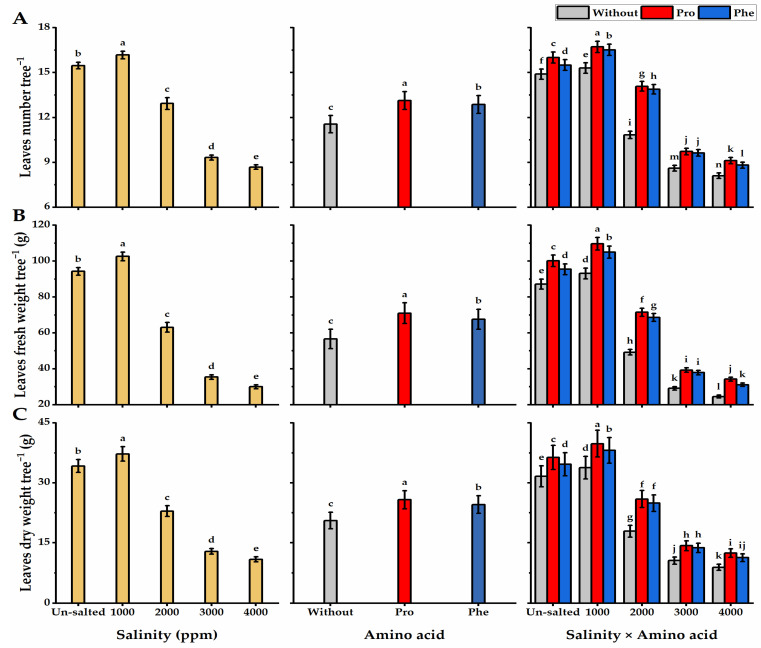
Influence of exogenous spray treatments of proline (Pro) or phenylalanine (Phe) on (**A**) leaves number, (**B**) leaves fresh weight, and (**C**) leaves dry weight tree^−^^1^ of moringa trees cultivated under different levels of salinity. Data are mean value ± SE. Bars with different letters are significantly different at *p* ≤ 0.05 level.

**Figure 3:**
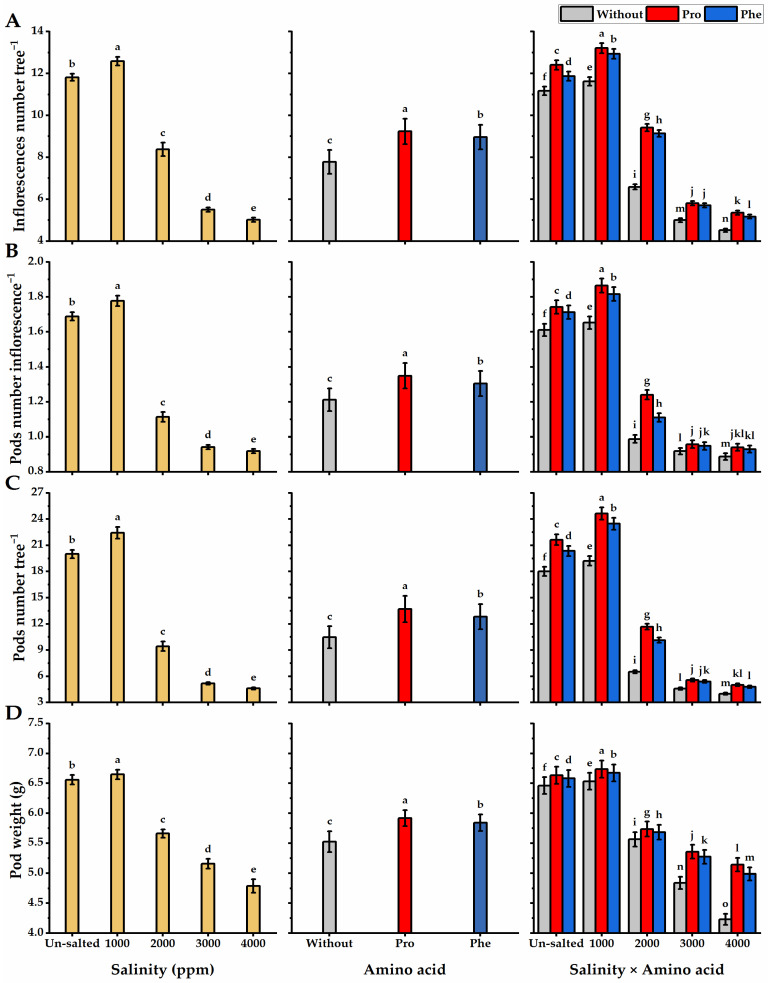
Influence of exogenous spray treatments of proline (Pro) or phenylalanine (Phe) on (**A**) inflorescences number tree^−^^1^, (**B**) pods number inflorescence^−^^1^, (**C**) pods number tree^−^^1^, and (**D**) pod weight of moringa trees cultivated under different levels of salinity. Data are mean value ± SE. Bars with different letters are significantly different at *p* ≤ 0.05 level.

Salinity applications negatively affected *moringa* height and stem diameter, as the least values (104.9 cm and 21.4 mm for plant height and stem diameter, respectively) were obtained following the highest level of salinity (4000 ppm), while 1000 ppm salinity-plants significantly exhibited the tallest trees (138 cm) with thicker stems (35.5 mm) (Figure 1). The height and stem diameter of moringa trees were significantly boosted following foliar application with Pro or Phe relative to without AA plants. Regarding the interaction, the tallest trees with thicker stems were obtained by Pro-plants cultivated under the lowest level of salinity (143 cm and 37.1 mm for plant height and stem diameter, respectively). However, the least values in this respect were obtained by without AA plants exposed to 4000 ppm saline conditions. When it comes to the leaf number as well as fresh and dry weights of the leaf, higher salinity treatments significantly resulted in a great decline in the leaf values, where the least leaf number and weights were gained when the highest salinity level (4000 ppm) was applied. Pro-treatments significantly improved leaf number, and their fresh and dry weights relative to Phe-application and without AA plants (Figure 2). Pro-application significantly exhibited the highest leaf number (13.1) with the heaviest weights per tree (70.9 and 25.7 g for fresh and dry weight respectively). In terms of the interaction, the Pro-plants grown under 1000 ppm of saline conditions significantly gave the maximum number of leaves (16.7) with the highest weights (109.6 and 39.8 g for fresh and dry weights, respectively) against the lowest values, which resulted from without AA plants and received 4000 ppm of salinity. The inflorescences and pod traits (inflorescences number tree^−1^, pods number inflorescence^−^^1^, pods number tree^−^^1^, pod weight, and yield of mature pod) of moringa plants were significantly boosted by Pro-application as compared with Phe-application (Figure 3). Concerning salinity treatments, increasing salinity levels from 2000 to 4000 ppm significantly caused a gradual reduction in the inflorescences and pod values. The values of inflorescences and pods were higher with Pro-treatments under 1000 ppm salinity than with without AA plants under the highest level of salinity.

**Figure 4:**
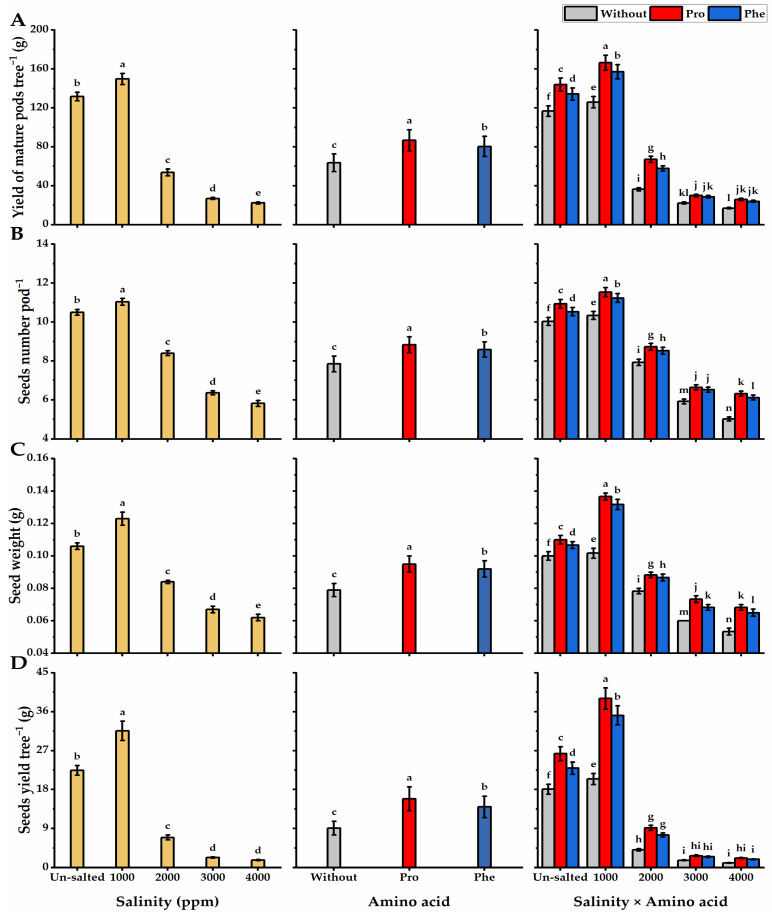
Influence of exogenous spray treatments of proline (Pro) or phenylalanine (Phe) on (**A**) yield of mature pods tree^−^^1^, (**B**) seeds number pod^−^^1^, (**C**) seed weight, and (**D**) seed yield tree^−^^1^ of moringa trees cultivated under different levels of salinity. Data are mean value ± SE. Bars with different letters are significantly different at *p* ≤ 0.05 level.

Pro-treatment significantly improved seed characteristics (seed number per pod^−^^1^, seed weight, and seed yield per tree^−^^1^) compared to Phe-treated and without AA plants (Figure 4). Concerning salinity treatments, a significant and gradual reduction in the values of seed number pod^−^^1^, seed weight, and seed yield was observed with increasing salinity levels from 2000 to 4000 ppm.

Pro-treatments produced the highest values of seed number per pod^−^^1^, seed weight, and seed yield per tree^−^^1^ at the lowest level of salinity (1000 ppm), while without AA plants they produced the lowest values at the highest level of salinity.

### 3.2. Total Chlorophyll

**Figure 5:**
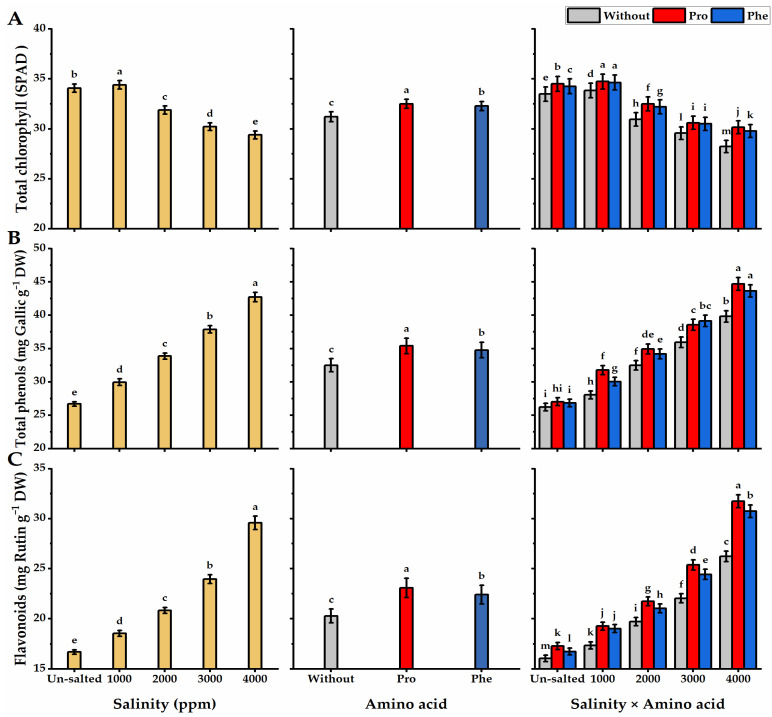
Influence of exogenous spray treatments of proline (Pro) or phenylalanine (Phe) on (**A**) total chlorophyll, (**B**) total phenols, and (**C**) flavonoids content of moringa leaves cultivated under different levels of salinity. Data are mean value ± SE. Bars with different letters are significantly different at *p* ≤ 0.05 level.

Total chlorophyll was significantly affected by amino acids and salinity applications (Figure 5). In this regard, chlorophyll content showed a negative correlation with salinity level, as its values decreased significantly at higher salinity levels, reaching the lowest levels (29.4 SPAD) at 4000 ppm salinity. The highest values of chlorophyll were followed by Phe-treatments, but the lowest values were produced by without AA plants. The plants subjected to Pro foliar spray and grown under 1000 ppm saline conditions significantly produced the highest values in this respect (34.7 SPAD).

### 3.3. Polyphenols Content

Increasing salinity levels led to an excess in polyphenol values, reaching their highest in the plants that received the highest salinity level (Figure 5). Total phenols and flavonoid content in moringa leaves were stimulated significantly as affected by Pro or Phe applications relative to without AA plants, as Pro-treatments significantly gave the highest polyphenol levels. The maximum levels of polyphenols (44.7 mg GAE g^−^^1^ DW and 31.8 mg RUT g^−^^1^ DW for phenols and flavonoids, respectively) were noticed in *moringa* plants that were foliar sprayed with Pro and grown under higher salinity levels. However, the lowest polyphenol levels (26.2 mg GAE g^−^^1^ DW and 16.1 mg RUT g^−^^1^ DW for phenols and flavonoids, respectively) were obtained from without AA plants grown under non-saline conditions.

### 3.4. Osmoprotectants Responses

**Figure 6:**
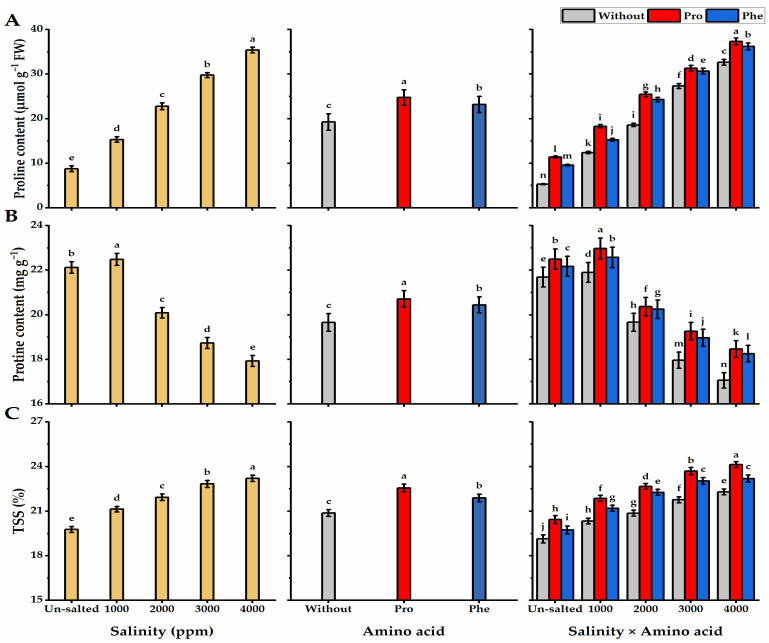
Influence of exogenous spray treatments of proline (Pro) or phenylalanine (Phe) on (**A**) proline, (**B**) protein, and (**C**) total soluble solids (TSS) of moringa leaves cultivated under different levels of salinity. Data are mean value ± SE. Bars with different letters are significantly different at *p* ≤ 0.05 level.

Results in Figure 6 show osmoprotectant concentrations in terms of proline, TSS, and protein, as influenced by amino acids and salinity applications. There was a positive and significant correlation between Pro and TSS content and salinity levels, as Pro and TSS levels significantly increase following salinity level increase, while the correlation between protein and salinity levels was negative. Foliar application with Pro significantly increased Pro, TSS, and protein concentrations in moringa leaves, followed by Phe-treatment. Regarding the interaction effect, the maximum Pro and TSS values (37.3% and 24.1% for Pro and TSS, respectively) were noticed in the leaves of Pro-treated trees cultivated under higher salinity levels, while the lowest Pro and TSS values (5.3% and 19% for Pro and TSS, respectively) were given by the without AA plants grown under the non-saline conditions. On the other hand, higher protein content was given by Pro-plants grown under 1000 ppm salinity (22.9 mg g^−^^1^), but this value decreased gradually with increasing salinity level to reach 18.5 mg g^−^^1^ under the same treatment.

### 3.5. Antioxidant Activity

**Figure 7:**
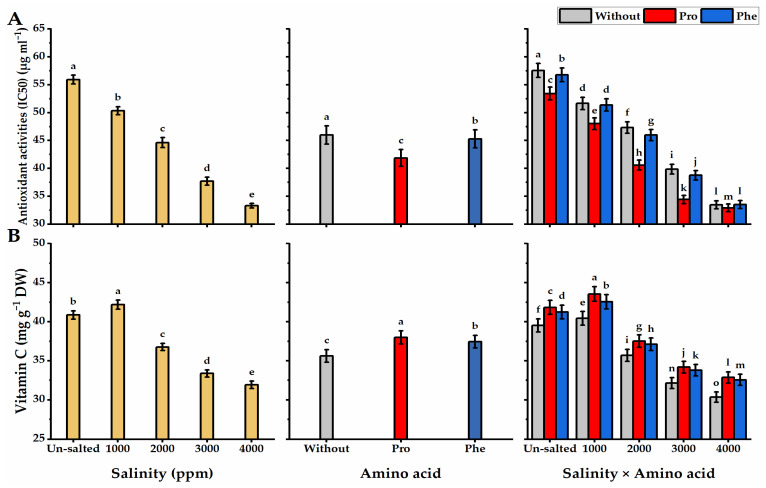
Influence of exogenous spray treatments of proline (Pro) or phenylalanine (Phe) on (**A**) antioxidant activity and (**B**) vitamin C of moringa trees cultivated under different levels of salinity. Data are mean value ± SE. Bars with different letters are significantly different at *p* ≤ 0.05 level.

The results presented in Figure 7A demonstrate the influence of Pro and Phe applications on the antioxidant activity produced in *moringa* leaves exposed to salinity stress. A remarkable increase in antioxidant activity resulted from salinity levels. A gradual and significant improvement in the antioxidant content was noticed with an elevation in salinity level, to reach the highest values under the highest level of salinity (55.9 µg mL^−^^1^), the opposite to the lowest antioxidant activity accumulated in unsalted *moringa* leaves (33.3 µg mL^−^^1^). The activity of antioxidants in moringa leaves has been increased following Pro and Phe-foliar applications. The maximum antioxidant activity was recorded with the Pro-plants exposed to the higher salinity level (57.6 µg mL^−^^1^). However, the lowest antioxidant activity was given by without AA plants cultivated under non-saline conditions (32.9 µg mL^−^^1^).

### 3.6. Vitamin C

The concentration of vitamin C was significantly impacted by Pro or Phe and salinity applications (Figure 7B). In this regard, vitamin C content was negatively correlated with salinity levels, as vitamin C content significantly declined at the higher levels of salinity, reaching the lowest value under 4000 ppm salinity (31.9 mg g^−^^1^ DW). The Pro-applied plants exhibited the highest values of vitamin C, but the lowest values were produced by without AA plants (35.6 mg g^−^^1^ DW). The plants subjected to Pro foliar spray and grown under non-saline conditions significantly produced the highest values in this respect (41.8 mg g^−^^1^ DW).

### 3.7. Mineral Content

**Figure 8:**
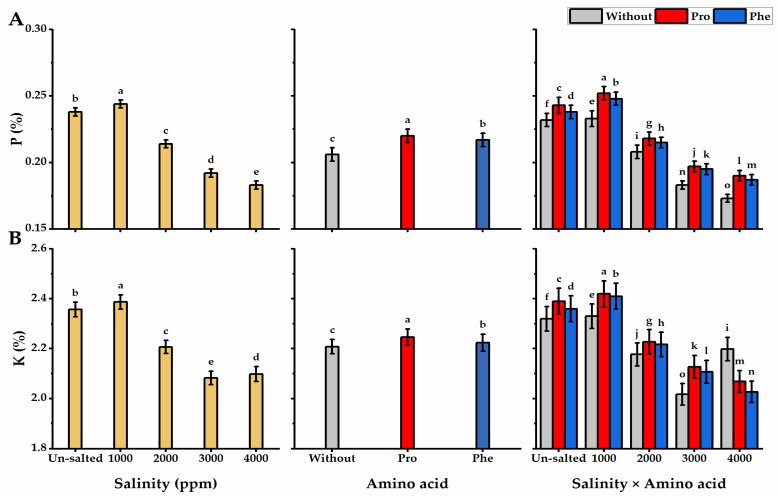
Influence of exogenous spray treatments of proline (Pro) or phenylalanine (Phe) on (**A**) phosphorus (P) and (**B**) potassium (K) content of moringa leaves cultivated under different levels of salinity. Data are mean value ± SE. Bars with different letters are significantly different at *p* ≤ 0.05 level.

The concentration of P and K was significantly impacted by Pro or Phe and salinity applications (Figure 8). In this regard, P and K levels were negatively correlated with salinity levels, as their values significantly declined at the higher levels of salinity, reaching the lowest value under 4000 ppm salinity. The Pro-applied plants exhibited the highest values of minerals, but the lowest values were produced by without AA plants. The plants subjected to Pro foliar spray and grown under non-saline conditions significantly produced the highest values in this respect.

### 3.8. Oil Percent

**Figure 9:**
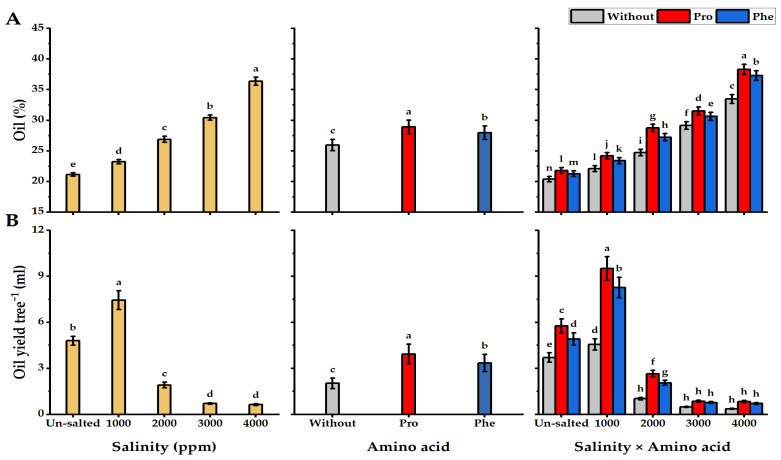
Influence of exogenous spray treatments of proline (Pro) or phenylalanine (Phe) on (**A**) oil % and (**B**) oil yield of moringa trees cultivated under different levels of salinity. Data are mean value ± SE. Bars with different letters are significantly different at *p* ≤ 0.05 level.

The oil % in moringa seeds gradually increased with increasing salinity levels (Figure 9A), as the highest oil % was obtained by moringa plants grown under the higher level of salinity (36.4%). Oil % significantly enhanced following amino acid applications, as Pro-foliar application significantly enhanced oil % in moringa seeds than in Phe and without AA plants. Moringa plants subjected to Pro-application and cultivated under higher salinity levels significantly exhibited the maximum oil % (38.3%). On the other hand, the lowest oil % was obtained from without AA plants cultivated under non-saline conditions (20.4%).

### 3.9. Oil Yield

Plants treated with the lowest level of salinity exhibited a significant increase in the oil yield tree^−^^1^ (7.4 mL) but increasing the salinity level from 1000 ppm to 4000 ppm led to a great decline in the oil yield obtained (0.64 mL) (Figure 9B). Foliar application with Pro or Phe positively promoted oil production in moringa seeds, and Pro-plants showed superiority over Phe and without AA plants in this respect. Concerning the interaction, Pro-plants cultivated under the lowest level of salinity (1000 ppm) significantly gave the maximum oil yield tree^−^^1^ (9.5 mL) relative to other treatments. On the other hand, the lowest oil yield tree^−^^1^ was given without AA plants grown under the higher salinity level (0.36 mL).

### 3.10. Fatty Acid Compositions

Results of fatty acid composition in moringa oilseeds produced under salinity and amino acids application indicated that all of the fatty acid were influenced by salinity and applied treatments (Table 3).

Under the lowest level of salinity, all fatty acids showed increments in their values, and these improvements have been increased following Pro and Phe applications than in without AA plants. Increasing salinity stress levels exhibited an increase in (C18:0), palmitic (C16:0), eicosenoic (C20:2), and behenic acids (C22:0), along with a reduction in linoleic (C18:2), and linolenic (C18:3) acids. There was a negative correlation between saturated and unsaturated fatty acid under saline stress conditions, indicating that enhancing one of them will cause a reduction in the other one. Amino acid application revealed an enhancement in unsaturated fatty acid reduction under salinity stress.

## 4. Discussion

Salinity stress is one of the detrimental stress factors causing considerable production declines in all crop plants. In the current study, moringa trees grown under the lowest level of salinity (1000 ppm) produced plants with better growth and more leaves and seed yield. Moringa trees are fast-growing evergreen or deciduous trees that can reach a height of 10 to 12 m [35]. Plants with faster growth presented a level of salinity tolerance because of the dilution effect, which lowered the negative impacts of excessive Na^+^ ions [36]. The rapid growth helps plants avoid salt stress and gives regular growth, causing higher biomass and yield. Therefore, the growth response can be used efficiently as an indicator to assess plant-stress tolerance [37]. Al-Hattab [14] stated that a gradual increase in the growth of the pepper plant was observed with increasing salinity levels, owing to the adaptive responses of the cells to the high levels of salinity via osmotic pressure regulation. On the other hand, the high levels of salinity depressed the growth and productivity of moringa trees, which resulted in a reduction in plant height, stem diameter, leaf weights, and inflorescence traits. Salinity slows physiological and metabolic activities and depresses cell growth and division [38]. Salt stress negatively affects the growth and productivity of plants through its osmotic impact, as the increase in the salt level in the soil solution is accompanied by a reduction in the water potential in the soil, preventing water absorption by the roots and causing a plant water deficit [39,40,41]. Furthermore, salt stress decelerates cell expansion and decreases the intracellular turgor [42,43]. Moreover, water deficiency negatively impacts the stomatal conductance, leading to a reduction in carbon fixation and assimilation and the amount of biomass yielded [44]. In this study, higher levels of salinity produced a marked decrease in the chlorophyll content of moringa leaves (Figure 3). Salinity stress stimulated chlorophyllase degraded enzymes and lowered nitrogen uptake, resulting in a reduction in chlorophyll content, photosynthetic rate, and depressed plant health and productivity [45,46]. Salt stress has been reported as a limiting factor for the growth and biomass yield of tomato [47], cotton [48], sugar beet [49], sweet pea [8], and bean [50] plants. In contrast, Pro and Phe treatments enhanced the plant growth and development of moringa trees under both saline and non-saline conditions. Salt stress tolerance contributes to an increase in osmoprotectant and antioxidant activity [13]. The osmoprotectants are active compounds accumulated in plants under osmotic pressure, depressing the osmotic potential and ameliorating the harmful effects of abiotic stress [10]. Their primary function is to maintain cell turgor, which improves water uptake, to maintain cell membranes and protein stability, and to act as free-radical scavengers [51,52]. Proline is one of the osmolyte compounds that accumulates under stress conditions. In this study, Pro and Phe foliar applications were followed by an enhancement in proline, TSS, and protein levels in moringa leaves. Such findings were reported by [21], who stated that Pro and Phe foliar applications increased proline, saccharides, and protein levels in maize and beans grew under saline conditions. Woody and herbaceous plants use Pro and Phe as an essential N and energy source [15,20]. Proline accumulation is one of the adaptation mechanisms against abiotic stress in plants [8]. It has an important role in protecting protein and chlorophyll from degradation and enhancing many enzyme activities [17]. Under salinity, proline maintained the efficiency of photosynthetic pigments and increased chlorophyll biosynthesis in onion leaves [53]. Under saline conditions, chilli pepper overproduced proline, phenylalanine, and ascorbic acid [14]. In this study, the enhancement in TSS content in moringa leaves following Pro and Phe applications was an indicator of better growth, leaves, seeds, and oil production, and more dry matter production and accumulation. Under saline conditions, TSS accumulated in plant cells, has an osmoprotective function that increases water uptake and nutrients required for improving metabolic activities and increases photosynthesis and metabolites [54]. Amino-acids-derived carbon enhances the plant growth via increasing the biomass conversion per plant N [55], and Pro shows supremacy over Phe in this respect, as Pro-treatment showed more biomass accumulation than Phe-application (Figure 2). This may be owing to the role of phenylalanine in stimulating more N and carbon allocation to the plant roots than shoots [20].

Plants that are more salt stress-tolerant have a stronger antioxidant defense system, which reduces ROS production and boosts the plants’ capacity to survive under stressful conditions [56,57]. Higher antioxidant defense is linked to better ROS scavenging. Moringa trees exposed to higher levels of salinity exhibited an increase in non-enzymatic antioxidants (vitamin C, phenolic, and flavonoid compounds) and antioxidant activity. Such results were obtained by [4,53]. Under stress conditions, ascorbic acid stimulates the activities of many enzymes and minimizes the harmful effects of oxidative stress through its synergic action with other antioxidants [56]. In stressed plants, a higher content of ascorbic acid means greater protection against oxidative damage. Leaves’ ascorbate content is positively correlated with salt stress levels [58,59]. Phenolic compounds are over-generated under salinity conditions to protect plant cells against the oxidative damage that occurs when affected by salts [60]. The phenolic compounds’ chemical structures help them to deactivate the singlet oxygen and have an important role as hydrogen donors, allowing them to scavenge ROS [54,61]. Pro and Phe-treated moringa presented higher antioxidant activity than without AA plants. Pro increased antioxidant activities, which reduced membrane damage, improved ROS detoxification, and preserved plasma membrane stability [62,63].

Salt stress negatively affects plant growth and development through the imbalance of minerals and specific ion toxicity (salt stress) and/or soil solution with low osmotic potential (water stress) [13,64]. Sodium and chloride elements are extremely harmful to plant life, especially in woody plants, due to their role in photosynthesis restrictions [65]. Higher salinity levels inhibit root development, as well as water and nutrient absorption [66]. Salt stress treatments led to a depression in P and K content in the leaves of moringa plants. Under salinity stress, excessive Na restricts the other essential elements absorbed [67]. Additionally, Na inhibits K absorption due to the competition between Na and K on the binding sites [68]. Pro and Phe applications presented more P and K concentrations in moringa leaves than in without AA leaves. Under salinity stress, Pro and Phe increased P and K concentrations in plant cells [69]. Amino acid applications lowered Na content in plant cells and boosted the macronutrient concentration under salt stress [21].

Increasing salinity levels resulted in a reduction in oil yield from moringa trees while increasing oil percent. This could be because, under stress conditions, plants shift their metabolites to generate secondary metabolites (osmolytes, fatty acids, antioxidant compounds, and terpenoids [35,70], with a greater reduction in plant growth and seed yield, resulting in an increase in oil percent with a reduction in seed yield. The larger drop in seed yield relative to oilseed yield is constant with that obtained by [35,71].

In the present study, all fatty acids were impacted by salinity stress and amino acid applications. The fatty acid composition of high-oleate cultivars was impacted by increasing saline levels, resulting in a lower oleic acid concentration [72]. The active oxygen species accumulated in plant cells under saline conditions deform the cellular redox systems, generate oxidative stress, and result in lipid peroxidation [73]. Lipid peroxidation affects membrane permeability and fluidity, causing lipid bilayer malfunction. Plant membranes are rich in linoleic and linolenic acids, which are important substrates for lipoxygenase [71]. Salinity stress exhibited an increase in saturated fatty acids (stearic, palmitic, eicosenoic, and behenic acids) accompanied by a reduction in polyunsaturated fatty acids (linoleic and linolenic acids). Saturated fatty acid increments may be due to lowering desaturase activity that occurs as an adaptive response to salt stress [74], as plants can protect their cells against the harmful oxidative impacts of salt ions via restructuring cell membranes with minimal polyunsaturated fatty acids [75]. Low unsaturation degree hampered membrane fluidity [76,77] and Na and Cl ion permeability [78].

## 5. Conclusions

*Moringa oleifera* plants supplemented with the lowest salinity level (1000 ppm) exhibited a significant enhancement in the growth and productivity. However, increasing salinity levels resulted a depression in their growth and development. Foliar applications with proline and phenylalanine significantly ameliorate the harmful effects of salinity. In this regard, proline treatments decelerated chlorophyll and protein degradation and exhibited higher osmoprotectants, vitamin C, total phenols, flavonoids, and antioxidant activity.

## Figures and Tables

**Table 1:** The physical and chemical properties of the experimental soil.

Property							
Sand (%)	29	OM	1.5	Soluble Ions (meqL^−1^)			
Silt (%)	38	CaCO_3_ (%)	1.6	HCO_3_^−^	2.37	Ca^+2^	6.02
						Mg^+2^	10.9
Clay (%)	33	pH	7.7	Cl^−^	9.27	Na^+^	4.42
Texture class	Clay loam	ECe (ds m^−1^)	2.26	SO_4_^−2^	10.59	K^+^	0.32

**Table 2:** Chemical characteristics of irrigation water used for the present study.

Parameter	pH	EC	Ca^+2^	Mg^+2^	Na^+^	K^+^	HCO^−3^	Cl^−^	SO_4_^−2^
		ds m^−1^	meqL^−1^						
Value	7.42	0.43	1.1	0.56	2.51	0.21	0.12	2.9	1.23

**Table 3:** Effect of proline (Pro) or phenylalanine (Phe) foliar applications on the chemical composition of the oil of *Moringa olivera* L. trees cultivated under different levels of salinity. Values are the mean of three triplicates ± SE.

Treatments	The Relative Percentage of the Fatty Acids (%)										Total Identified	Total Unsaturated Fatty Acids	Total Saturated Fatty Acids
	Stearic Acid	Oleic Acid	Linoleic Acid	α-Linolenic Acid	Palmitic Acid	Palmitoleic Acid	Eicosenoic Acid	Paullinic Acid	Behenic Acid	Lignoceric Acid			
	18:00	18:01	18:02	18:03	16:00	16:01	20:00	20:01	22:00	24:00			
T1	3.08 ± 0.05	65.37 ± 1.04	3.17 ± 0.05	0.71 ± 0.01	3.83 ± 0.06	2.30 ± 0.04	1.90 ± 0.03	2.59 ± 0.04	3.83 ± 0.06	0.85 ± 0.01	87.64	74.13	13.50
T2	3.39 ± 0.05	66.69 ± 1.07	3.23 ± 0.05	0.73 ± 0.01	4.01 ± 0.06	2.50 ± 0.04	1.95 ± 0.03	2.70 ± 0.04	3.95 ± 0.06	0.87 ± 0.01	90.01	75.85	14.16
T3	3.40 ± 0.05	66.21 ± 1.06	3.19 ± 0.05	0.72 ± 0.01	4.04 ± 0.06	2.42 ± 0.04	1.98 ± 0.03	2.67 ± 0.04	4.01± 0.06	0.88 ± 0.01	89.51	75.21	14.31
T4	3.65 ± 0.06	68.12 ± 1.09	3.39 ± 0.05	0.76 ± 0.01	4.17 ± 0.07	2.63 ± 0.04	2.09 ± 0.03	2.85 ± 0.05	4.16 ± 0.07	0.91 ± 0.01	92.73	77.74	14.99
T5	3.78 ± 0.06	68.88 ± 1.12	3.57 ± 0.06	0.77 ± 0.01	4.03 ± 0.07	2.89 ± 0.05	2.15 ± 0.03	3.00 ± 0.05	4.00 ± 0.07	0.00 ± 0.01	93.07	79.11	13.96
T6	3.76 ± 0.06	68.03 ± 1.11	3.39 ± 0.06	0.75 ± 0.01	4.19 ± 0.07	2.75 ± 0.04	2.15 ± 0.03	2.99 ± 0.05	4.24 ± 0.07	0.89 ± 0.01	93.13	77.91	15.22
T7	3.98 ± 0.06	62.02 ± 0.99	3.01 ± 0.05	0.65 ± 0.01	4.39 ± 0.07	2.13 ± 0.03	2.31 ± 0.04	2.36 ± 0.04	4.50 ± 0.07	0.99 ± 0.02	86.36	70.18	16.18
T8	4.12 ± 0.07	63.32 ± 1.01	3.10 ± 0.05	0.69 ± 0.01	4.42 ± 0.07	2.24 ± 0.04	2.43 ± 0.04	2.42 ± 0.04	4.61 ± 0.07	1.04 ± 0.02	88.38	71.76	16.62
T9	4.16 ± 0.07	62.78 ± 1.00	3.07 ± 0.05	0.70 ± 0.01	4.44 ± 0.07	2.21 ± 0.04	2.47 ± 0.04	2.40 ± 0.04	4.65 ± 0.07	1.06 ± 0.02	87.93	71.16	16.77
T10	4.24 ± 0.07	59.68 ± 0.95	2.28 ± 0.04	0.57 ± 0.01	4.54 ± 0.07	1.92 ± 0.03	2.58 ± 0.04	2.09 ± 0.03	4.79 ± 0.08	0.00 ± 0.02	82.69	66.54	16.15
T11	4.38 ± 0.07	60.49 ± 0.97	2.89 ± 0.05	0.60 ± 0.01	4.66 ± 0.07	2.06 ± 0.03	2.69 ± 0.04	2.17 ± 0.03	5.08 ± 0.08	1.11 ± 0.02	86.12	68.21	17.91
T12	4.44 ± 0.07	60.35 ± 0.96	2.87 ± 0.05	0.59 ± 0.01	4.71 ± 0.08	2.01 ± 0.03	2.72 ± 0.04	2.14 ± 0.03	5.19 ± 0.08	1.12 ± 0.00	86.15	67.97	18.18
T13	5.01 ± 0.07	61.45 ± 0.89	2.29 ± 0.03	0.56 ± 0.01	5.37 ± 0.08	1.03 ± 0.01	3.29 ± 0.05	2.11 ± 0.03	6.06 ± 0.09	0.00 ± 0.00	87.24	67.50	19.74
T14	5.13 ± 0.07	63.85 ± 0.92	2.34 ± 0.03	0.60 ± 0.01	5.53 ± 0.08	1.19 ± 0.02	3.38 ± 0.05	2.20 ± 0.03	6.27 ± 0.09	1.14 ± 0.02	89.28	67.84	21.44
T15	5.09 ± 0.08	60.99 ± 0.92	2.31 ± 0.03	0.57 ± 0.01	5.37 ± 0.08	1.13 ± 0.02	3.40 ± 0.05	2.07 ± 0.03	6.13 ± 0.09	1.13 ± 0.02	88.19	67.07	21.12

T1; unsalted × without AA, T2; unsalted × Pro, T3; unsalted × Phe, T4; 1000 ppm × without AA, T5; 1000 ppm × Pro, T6; 1000 ppm × Phe, T7; 2000 ppm × without AA, T8; 2000 ppm × Pro, T9; 2000 ppm × Phe, T10; 3000 ppm × without AA, T11; 3000 ppm × Pro, T12; 3000 ppm × Phe, T13; 4000 ppm × without AA, T14; 4000 ppm × Pro, T15; 4000 ppm × Phe.

## Data Availability

The data presented in this study are available within the article.
